# Supplementary material for: The latitudinal variation in amphibian speciation rates revisited
Source: Commun Biol. 2025 May 28;8:822. doi: 10.1038/s42003-025-08225-2 (PMC12119841; doi:10.1038/s42003-025-08225-2)
Supplement: Supplementary file 1 — Supplementary Information [file 42003_2025_8225_MOESM1_ESM.pdf]

SUPPORTING INFORMATION for:

The latitudinal variation in amphibian speciation rates revisited

Adrián García-Rodríguez[1], Fabricio Villalobos[2], Julián A. Velasco[3], Franz Essl[1] and Gabriel C. Costa[4].

[1]Division of BioInvasions, Global Change & Macroecology, Department of Botany and Biodiversity Research, University of Vienna, Rennweg 14, Vienna 1030, Austria
[2]Red de Biología Evolutiva, Instituto de Ecología A.C., Xalapa, Veracruz CP 91073, Mexico
[3]Instituto de Ciencias de la Atmósfera y Cambio Climático, Universidad Nacional Autónoma de México, Ciudad Universitaria, CP 04510, Mexico City, Mexico
[4]Department of Biology and Environmental Sciences, Auburn University at Montgomery, Montgomery, AL 36117, United States

Supporting figures

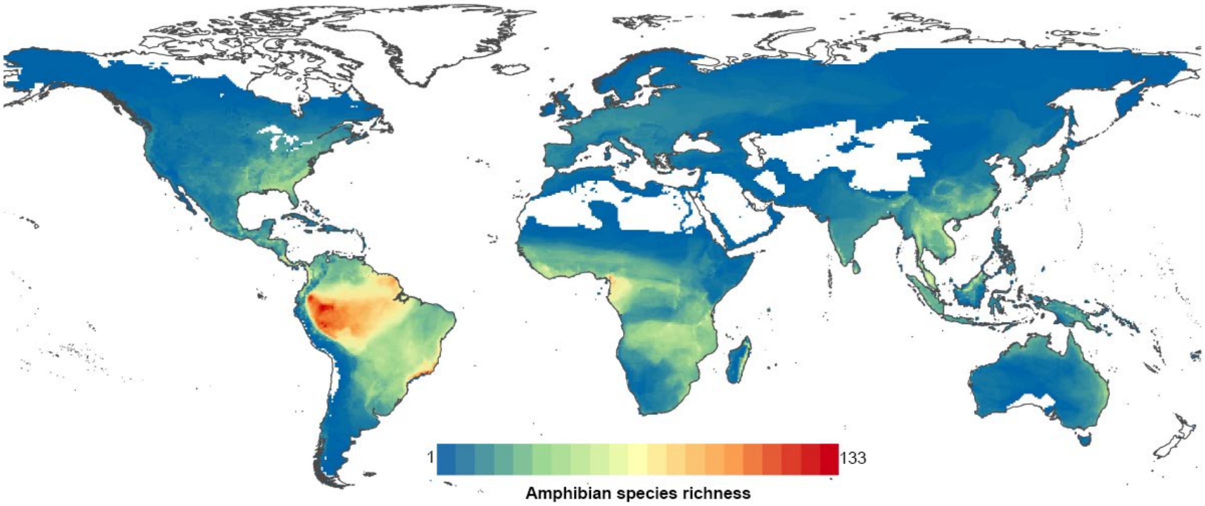

Supplementary Figure 1. Global patterns of species richness in amphibians, inferred from the distribution ranges of nearly 7000 species (~ 80% of the known amphibian diversity) at a 0.5-degree resolution (~50x50 km at the Equator).

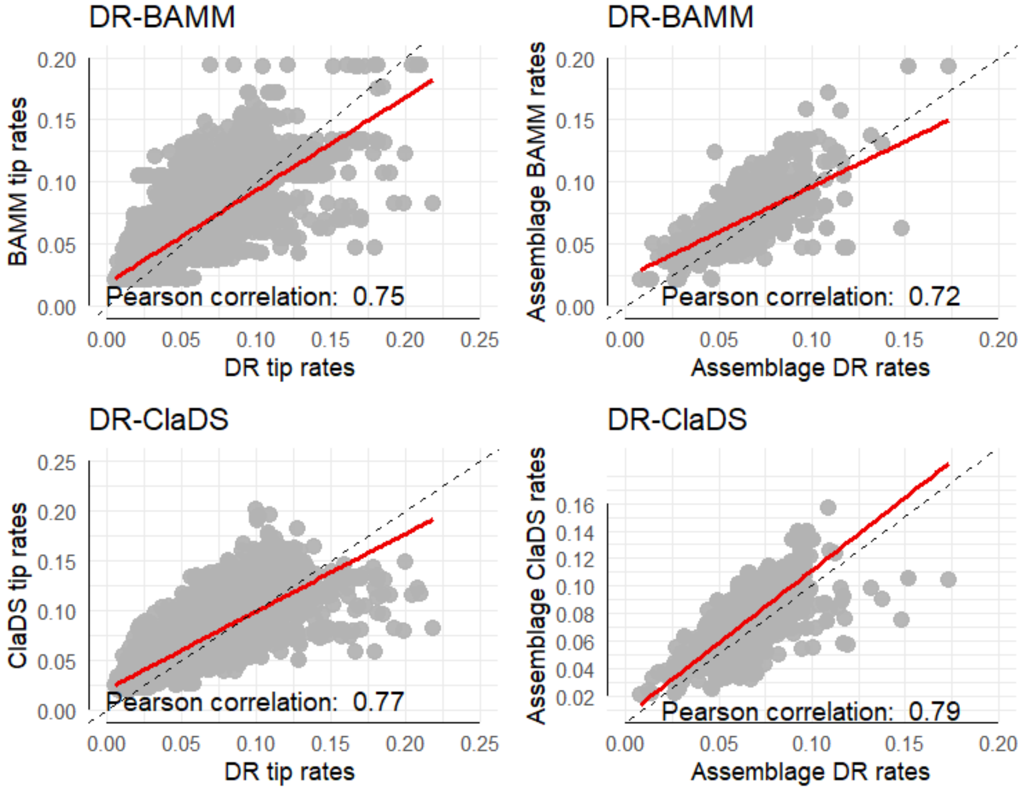

Supplementary Figure 2. Correlations between speciation estimates obtained using three different approaches. Plots on the left column show correlations between tip-level rates and plots on the right column depict correlations among assemblage mean rates.

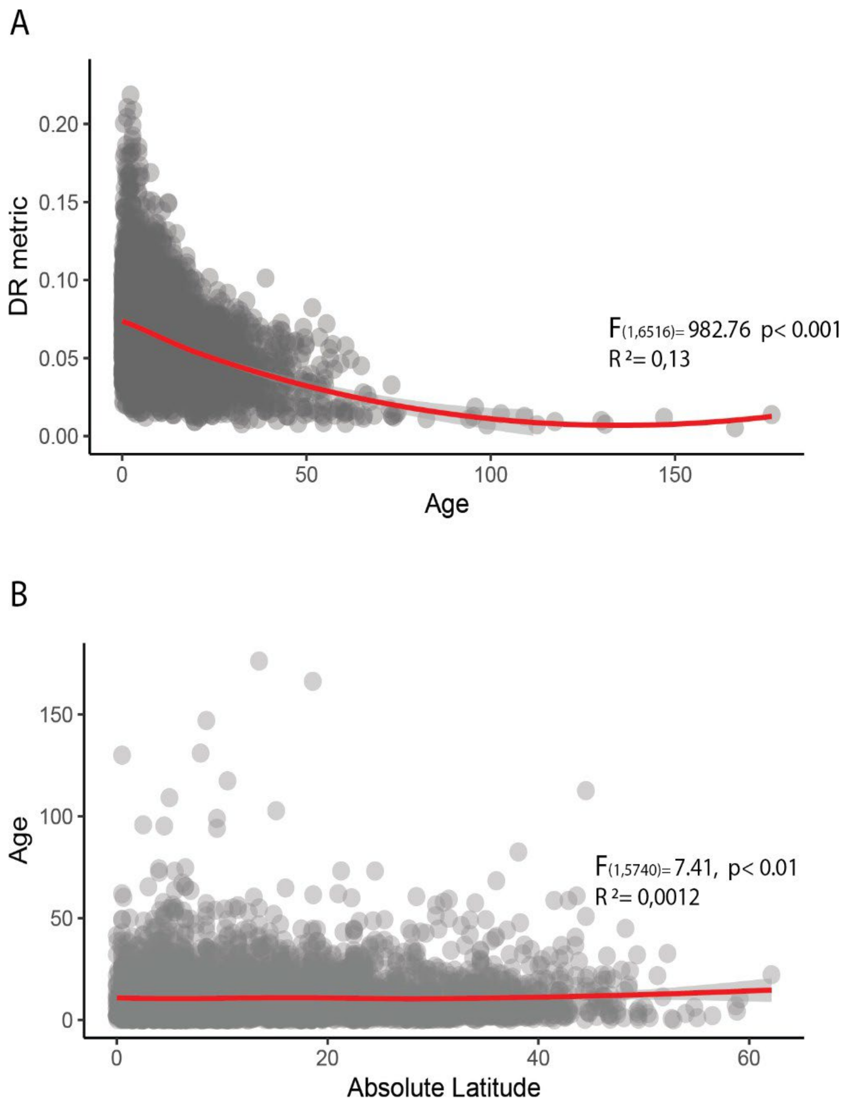

Supplementary Figure 3. Relationships among tip-level speciation rates, species age, and their respective latitudinal mid-point. **A.** Correlation between speciation rates and species age inferred from their respective branch lengths. **B**. Correlation between these ages and the mid- latitude of each species distribution range (right).

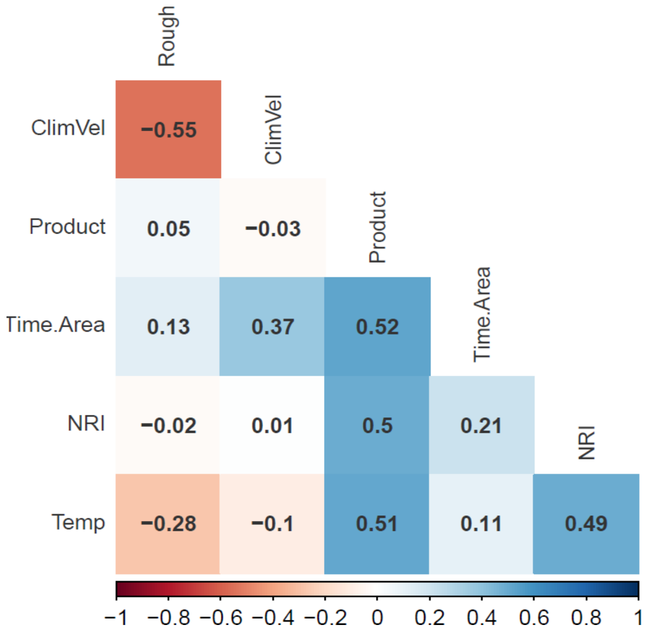

Supplementary Figure 4. Pearson correlations among predictors compiled from [1] and estimated in this work for the 32 bioregions studied.

Supporting tables.

Supplementary Table 1. Estimations of speciation rates and the predictors tested for each of the 32 bioregions studied. Values for area productivity and temperature were obtained from [1].

Bioregion	Mean Spec. Rate	Time Area (10^3 Km^2)	Productivity (g C m^{-2} y^{-1})	Temperature (°C)	NRI	Terrain roughness	Climatic velocity
Afrotropics Desert	0.055	12459.2	142.3	23.58	4.17	69.31	0.121
Afrotropics DryForest	0.053	158155.2	591.5	23.26	3.33	41.41	0.174
Afrotropics Mediterranean	0.049	480	524.1	16.78	4.59	153.38	0.033
Afrotropics TropMoist	0.05	1059974.31	887.88	23.7	9.79	63.72	0.195
Australia Desert	0.06	18522	179.36	21.89	5.7	16.31	0.194
Australia DryForest	0.063	25166.4	530.14	25.15	0.54	29.75	0.129
Australia Grasslands	0.064	2705.17	429.17	16.28	7.67	22.25	0.190
Australia Mediterranean	0.063	4015	467.12	16.7	7.01	22.25	0.076
Australia Temperate	0.062	110249.55	702.59	11.48	7.13	119.99	0.043
Eurasia Boreal	0.066	35864.47	338.12	-3.01	-0.08	73.08	0.203
Eurasia Desert	0.07	95851.6	130.58	16.1	2.05	53.48	0.175
Eurasia Grasslands	0.066	20311.97	298.75	4.56	-1.85	56.07	0.223
Eurasia Mediterranean	0.058	10260	502.4	14.76	-2.78	139.64	0.062
Eurasia Temperate	0.064	1005043.28	544.94	8.74	-4.98	122.21	0.199
Eurasia Tundra	0.063	45651.2	167.52	-9.47	-0.17	144.27	0.117
Indo-Malay DryForest	0.06	17460.8	600.79	26.17	8.9	66.18	0.189

Indo-Malay TropMoist	0.059	872028.17	917.1	24.74	11.39	164.1	0.134
Madagascar Desert	0.049	622.26	409.44	22.81	3.62	67.78	0.056
Madagascar DryForest	0.049	622.26	619.52	25.04	2.9	75.43	0.053
Madagascar TropMoist	0.051	31042.68	964.75	21.13	4.75	145.92	0.048
North America DryForest	0.065	58721.6	693.67	23.75	2.73	50.63	0.092
North America Boreal	0.078	71643.7	338.69	-2.04	-1.46	55.94	0.217
North America Desert	0.066	12059	264.96	16	-0.79	112.26	0.106
North America Grasslands	0.077	13310.66	490.72	10.14	-3.28	35.92	0.172
North America Mediterranean	0.073	605	370.24	14.46	0.69	171.21	0.030
North America Temperate	0.07	729410.74	549.88	8.15	-0.23	123.64	0.136
North America Tundra	0.072	47857.6	136.62	-8.2	-1.29	186.5	0.096
South America TropMoist	0.068	701314.44	946.52	24.12	16.26	94	0.239
South America Desert	0.068	6094	567.99	23.56	3.54	113.29	0.097
South America Grasslands	0.066	6998.37	482.98	13.34	14.51	41.81	0.111
South America Mediterranean	0.071	740	306.02	13.25	3.55	178.6	0.058
South America Temperate	0.08	156836.96	475.3	9.81	8	323.55	0.027

References

1. Jetz, W. & Fine, P. V. A. Global gradients in vertebrate diversity predicted by historical area-productivity dynamics and contemporary environment. *PLoS Biol* **10**, e1001292 (2012).
